# Spatially Coupled Cl_2_ Confinement and Activation at Curved Ni Single‐Atom Sites for Highly Reversible Li–Cl_2_ Batteries

**DOI:** 10.1002/anie.6857946

**Published:** 2026-07-31

**Authors:** Minggang Xie, Keren Jiang, Xuehai Tan, Xuesong Xie, Zheng Cheng, Xianbin Meng, Yuxuan Xue, Yi Guan, Zhixuan Chen, Yifan Li, Renfei Feng, Zhi Li

**Affiliations:** ^1^ Department of Chemical and Materials Engineering University of Alberta Edmonton Alberta Canada; ^2^ State Key Laboratory of Supramolecular Structure and Materials Jilin University Changchun China; ^3^ Canadian Light Source (CLS) Saskatoon Saskatchewan Canada

**Keywords:** Cl_2_ Management, curvature‐modulation, lithium–chlorine batteries, single‐atom catalysis, wide‐temperature operation

## Abstract

Leveraging the high‐potential Cl_2_/LiCl redox couple (∼3.6 V), rechargeable Li‐Cl_2_ batteries hold compelling promise for next‐generation energy storage. However, their practical viability is severely impeded by poor Cl_2_ retention and sluggish conversion kinetics, particularly under deep‐cycling and high‐rate regimes. Here, we present a spatially coupled Cl_2_‐management cathode that integrates Cl_2_ confinement and short‐range transport within a micropore‐rich matrix with spontaneous activation at dense pore wall‐anchored, curved Ni single‐atom sites. Via this closed‐loop pathway from Cl_2_ storage to LiCl deposition, the Li‐Cl_2_ battery achieves a remarkable 1.21 V polarization reduction at 1500 mAh g^−1^ cut‐off capacity at 2000 mA g^−1^. Consequently, an ultrahigh cumulative capacity exceeding 1.0 million mAh g^−1^, which doubles the lifespan of state‐of‐the‐art Li‐Cl_2_ systems, is achieved through the integrated approach incorporating this pathway, the robust, partially graphitized support, and an optimized discharge‐cutoff protocol. Furthermore, the exceptional all‐climate robustness (–40°C to +70°C), coupled with a high reversible areal capacity of 7.65 mAh cm^−2^ in pouch cells, establishes a new paradigm for practical, high‐energy storage systems.

## Graphical abstract

A robust cathode featuring curvature‐engineered Ni single‐atoms anchored within hierarchical pores effectively confines Cl_2_ and accelerates Cl^0^/Cl^−1^ redox kinetics. This synergistic architecture unlocks the potential of Li‐Cl_2_ secondary batteries with an unprecedented 1×10^6^ mAh g^−1^ cumulative capacity, all‐climate resilience (–40°C to + 70°C), and 7.65 mAh cm^−2^ pouch‐cell integration.

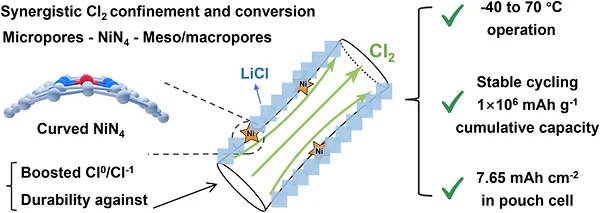

## 1 Introduction

The global shift toward high‐density, low‐carbon, and sustainable energy systems has imposed unprecedented demands on advanced energy storage technologies. Among the diverse energy storage systems, the primary Li–SOCl_2_ battery is distinguished by its ultra‐high energy density up to 710 Wh kg^−1^ [1], although its application has been confined to one‐time use for over three decades [2, 3]. Excitingly, in 2021, Dai et al. pioneered a coupled strategy that integrated TFSI^−^/FSI^−^ additives with highly microporous carbon cathodes, enabling reversible Cl_2_/Cl^−^ redox reactions, thereby opening the door to secondary Li/Na‐Cl_2_ batteries in Li/Na–SOCl_2_ systems [4]. Since this breakthrough, an increasing number of studies have broadened the scope of this emerging chemistry, uncovering important advances such as the additional redox pathways involving SCl_2_/S_2_Cl_2_ [5], the development of high‐energy‐efficiency SO_2_/SO_2_Cl_2_ batteries based on synergistic S‐Cl chemistry [6], the elucidation of the catalytic role of fluorinated electrolyte additives [7], the regulation of discharge‐product deposition behavior [8], iodine‐induced self‐depassivation [9], the design of wide‐temperature, noncorrosive organic electrolytes [10], and reversible Si‐anode redox [11], all of which highlight the great potential of Li/Na‐Cl_2_ batteries. Similar to other anion‐based redox systems, the Cl_2_/Cl^−^ redox at cathodes makes cathodes the primary determinant of overall performance in Li–Cl_2_ batteries. Encouragingly, to date, a growing variety of cathode materials have been explored, including crystalline porous frameworks such as MOFs [12] and COFs [13], porous polymers [14], and ICl_3_ cathodes [15].

Owing to their abundance, low cost, and lightweight nature, carbonaceous materials remain the most extensively investigated, featuring diverse structural designs such as defect engineering [16], bicontinuous structures [17], porosity enhancement [18], and blocking pore design [19], among others. Pioneering studies have established that porous carbon architectures, particularly those enriched with micropores are indispensable for Li‐Cl_2_ batteries due to their exceptional capability to physically confine Cl_2_ species. However, as the redox host, pristine carbon materials often lack intrinsic active sites, resulting in sluggish Cl_2_/Cl^−^ conversion kinetics. A typical observation is that discharge kinetics lag behind charging kinetics, thereby compromising Li‐Cl_2_ battery power density [4]. This imbalance originates from sluggish Cl_2_ to Cl^−^ conversion on conventional porous carbon cathodes with limited catalytic sites, leading to Cl_2_ escape and parasitic reactions at the Li anode that forms an insulating, irreversible LiCl layer and severely compromises cycling stability [19]. Single‐atom catalysts (SACs) with their unique electronic structures and maximum atom efficiency are a promising solution to address these kinetic limitations and suppress active species crossover [20, 21, 22]. A recent study on Co SACs for Li–Cl_2_ batteries showed that CoN_4_ centers lower the kinetic barrier for LiCl oxidation, underscoring the potential of SACs for chlorine redox [23]. Nevertheless, the high volatility of Cl_2_ requires effective local retention, whereas the sluggish LiCl/Cl_2_ conversion demands rapid interfacial kinetics to mitigate electrode passivation and Cl_2_ shuttling [24]. Thus, an integrated Cl_2_‐management architecture that spatially couples Cl_2_ confinement and LiCl deposition with efficient catalytic conversion sites is highly desirable but remains insufficiently developed. Furthermore, the Cl^0^‐rich oxidizing environment might exacerbate the oxidation of defect‐rich carbon hosts; such degradation, reflecting the known challenges in acidic oxygen electrocatalysis [25, 26], likely leads to compromised cycling durability.

In this research, we report a sustainable Ni‐mediated upcycling strategy for constructing a hierarchical porous cathode with anchored NiN_4_ sites, tailored for high‐performance Li‐Cl_2_ batteries. This configuration establishes a spatially coupled Cl_2_‐management architecture, in which abundant micropores (micropore volume fraction ∼ 50.1%) serve as local Cl_2_ reservoirs and enable short‐range redistribution, while densely distributed NiN_4_ sites anchored on the pore walls facilitate efficient LiCl/Cl_2_ conversion within the adjacent larger pores. Driven by this spatially coupled mechanism, the developed Li‐Cl_2_ battery demonstrates a remarkable polarization reduction under deep‐discharge conditions of 1500 mAh g^−1^ cut‐off capacity, exhibiting a 1.21 V decrease at 2000 mA g^−1^. Theoretical calculations also reveal that this curvature engineering stabilizes the NiN_4_ coordination while intrinsically facilitating reversible conversion between Cl_2_ and LiCl. Under stringent testing (1500 mA g^−1^ and 1500 mAh g^−1^ cut‐off capacity), the battery maintains stable cycling for 670 cycles, yielding a cumulative capacity exceeding 1.0 million mAh g^−1^, a twofold enhancement over state‐of‐the‐art Li‐Cl_2_ systems. This performance is further validated by the stable operation across a wide temperature window (–40 to +70°C) and the reversible areal capacity of 7.65 mAh cm^−2^ in pouch cells, underscoring its all‐climate and high‐energy applications.

## 2 Results and Discussion

The schematic in Figure 1a outlines a representative Li–Cl_2_ battery configuration employing Li metal as the anode and a porous carbon as the cathode. The first discharge process is the same as that of the primary Li‐SOCl_2_ battery, involving the reduction of SOCl_2_, yielding LiCl as the main product. In subsequent reversible cycles from charging to re‐discharging, the electrochemical behavior is dominated by the redox reaction between LiCl and Cl_2_ [4, 16]:

(1) $$2\mathrm{SOC}l_{2}+4Li^{+}+4e^{-}\to \;S+SO_{2}+4\mathrm{LiCl}$$

(2) $$2\mathrm{LiCl}-2Li^{+}-2e^{-}↔\;Cl_{2}$$

**FIGURE 1:**
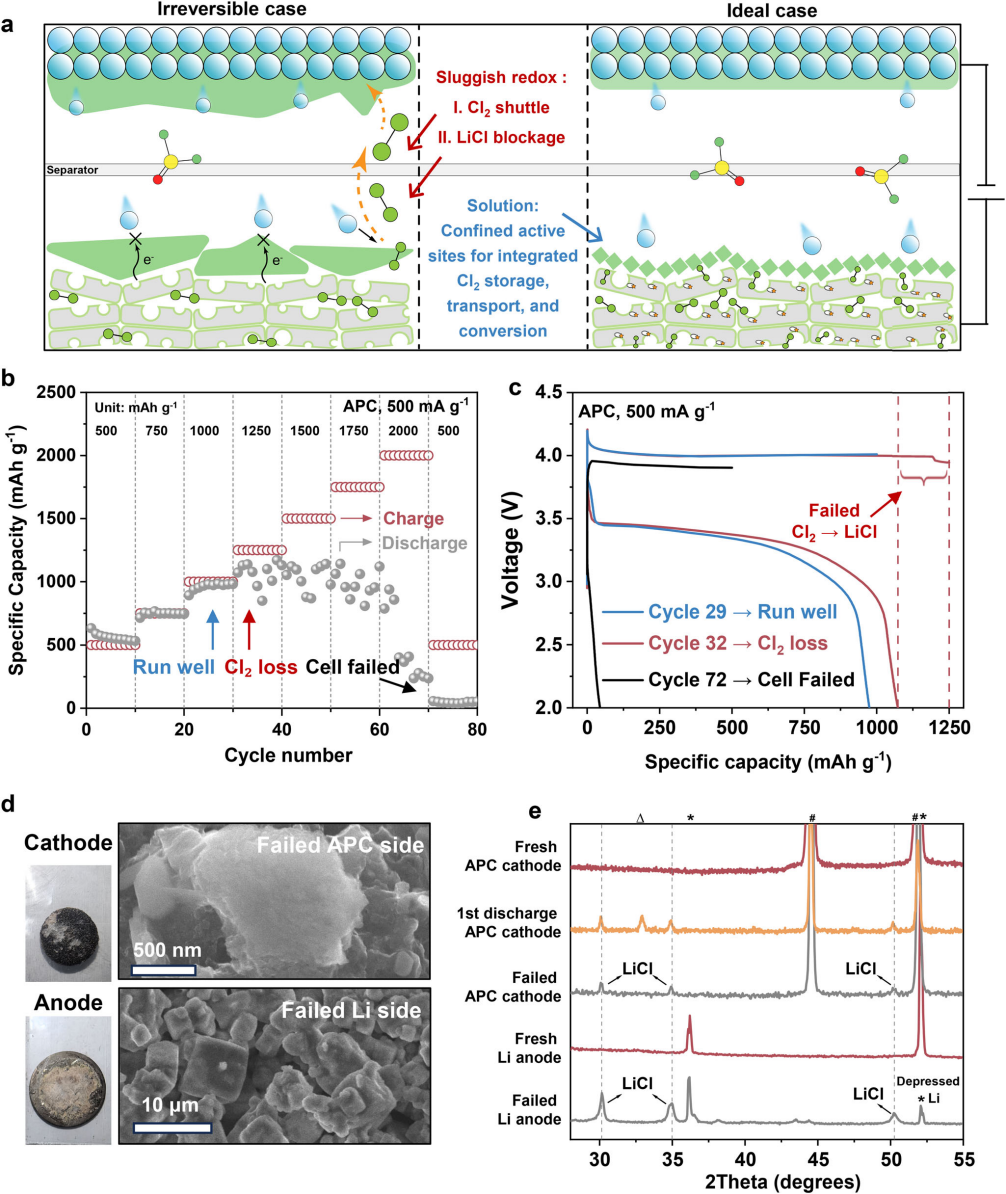
Failure mechanism of Li–Cl_2_ batteries under deep discharge. (a) Schematic illustration of the irreversible and ideal Li–Cl_2_ battery states: (left) Weak Cl_2_ retention and sluggish Cl_2_/Cl^−^ redox lead to Cl_2_ shuttling and insufficient LiCl deposition regulation. (right) Hierarchically porous networks with abundant micropores, functionalized with catalytic motifs to facilitate Cl_2_ retention, transport and redox kinetics. (b) Cycling performance of the Li‐Cl_2_@APC battery at 500 mA g^−1^ with cut‐off capacities stepped from 500 to 2000 mAh g^−1^ and returning to 500 mAh g^−1^. (c) Charge‐discharge curves of Li‐Cl_2_@APC with different states. (d) Optical and SEM images of the anode and cathode surfaces from the failed Li‐Cl_2_@APC battery. (e) XRD patterns of the anode and cathode at various states. The peaks corresponding to Li, Ni foam, and sulfur are marked with *, #, and Δ, respectively.

To elucidate the intrinsic challenges in Li–Cl_2_ batteries, we first employed a typical hierarchically porous cathode (denoted as APC), derived from asphaltene via Na_2_CO_3_ activation (synthesis and characterization detailed later). In line with the established protocol [9], after the initial discharge to 2.0 V (LiCl deposition, Equation (1)), the cell underwent galvanostatic cycling where it was charged to a specific capacity and then discharged to 2.0 V. These capacity limits were increased stepwise from 500 to 2000 mAh g^−1^ (10 cycles each) at 500 mA g^−1^ to determine the maximum reversible capacity (Figure 1b). Using this approach, Li–Cl_2_@APC achieved a maximum reversible capacity of 980 mAh g^−1^ at a 1000 mAh g^−1^ charge limit. Upon extending the charge to 1250 mAh g^−1^, the characteristic Cl_2_ evolution plateau was sustained at ∼4.0 V (Figure 1c), yet this deep charging failed to translate into a commensurate discharge capacity. Even worse, increasing the charging capacity to 2000 mAh g^−1^ caused severe cell failure, manifested as a negligible discharge capacity of only 55 mAh g^−1^ (11% CE) in the subsequent cycle. This irreversible degradation was primarily driven by weak Cl_2_ binding and sluggish kinetics on the APC cathode, leading to the diffusion of unbonded Cl_2_ across the separator to attack the Li anode [5]. This parasitic reaction continuously depleted active Li, leading to the formation of insulating bulk LiCl cubes with edge lengths exceeding 5 µm (Figure 1d), an observation further corroborated by the intense LiCl reflections and suppressed Li peaks in the XRD pattern (Figure 1e). Meanwhile, the substantial irreversible LiCl accumulation observed on the APC cathode (Figure 1e) was associated with the lack of catalytic sites, leading to increased polarization and further hindering the sluggish Cl_2_/LiCl redox [23]. Such degradation mechanisms underpin the broader challenge in the field, where the cycling durability of Li–Cl_2_ batteries was frequently hampered, often falling short of 500 cycles, particularly when subjected to high current densities and deep cycling. Consequently, the development of functional and robust scaffolds capable of both effective Cl_2_ retention and accelerated redox kinetics remains the key to unlocking durable Li‐Cl_2_ batteries.

By strategically leveraging the dual role of Ni species, which initially served as graphitization catalysts during synthesis [27, 28] and subsequently persisted as robust single‐atom sites for Cl_2_/LiCl redox conversion, we engineered a hierarchical porous carbon matrix to function as a highly durable and active cathode. As illustrated in Figure 2a, a precursor mixture of asphaltene, NiPc, and Na_2_CO_3_ was subjected to ball‐milling, annealing, and acid washing. The resulting product, denoted as NiN_4_‐cPC, featured atomically dispersed Ni embedded in a curved, porous graphitic carbon framework. Scanning and transmission electron microscopy (SEM/TEM) revealed that NiN_4_‐cPC possessed a sponge‐like morphology composed of assembled nanosheets, forming a 3D hierarchical network (Figure 2b,c). This multiscale architecture was characterized by a homogeneous distribution of C, N, and Ni throughout the matrix (Figure 2d), without discernible metal‐containing nanoparticles. Further investigation via high‐resolution TEM (Figure 2e) revealed a sophisticated hierarchy, with pronounced cylindrical‐like micropores intricately embedded within the larger macropore walls. This multiscale organization is expected to facilitate electrolyte infiltration, provide space for LiCl deposition, promote local Cl_2_ confinement and shorten diffusion pathways, thereby suppressing the shuttle effect and improving Cl_2_ utilization.

**FIGURE 2:**
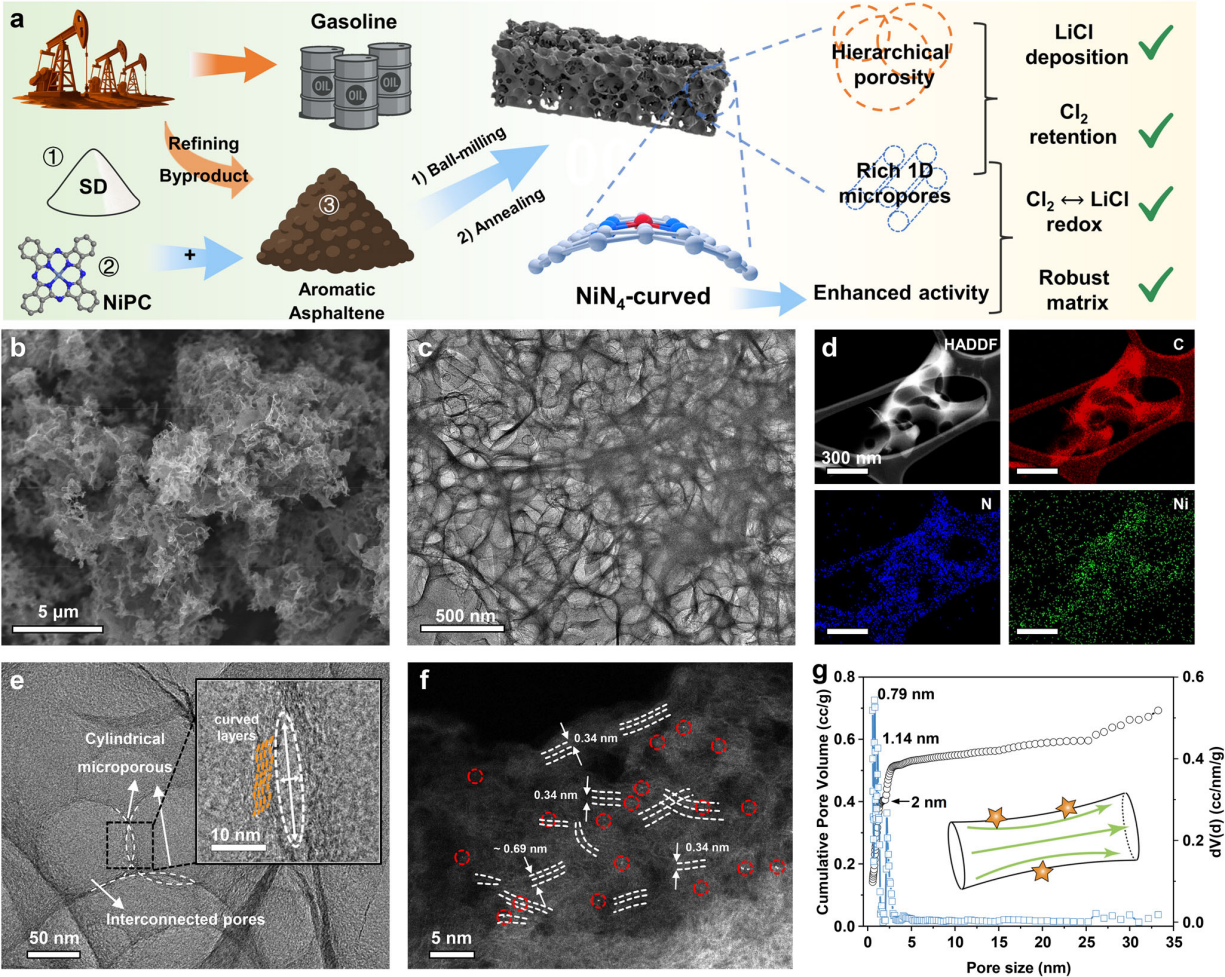
Synthesis strategy and structural characterization of NiN_4_‐cPC. (a) Schematic illustration of the preparation procedure for NiN_4_‐cPC and its design rationale for Li‐Cl_2_ batteries. (b) SEM, (c, e) TEM, and (d) HAADF‐STEM images with the corresponding elemental mappings of C, N, and Ni. (f) Aberration‐corrected HAADF‐STEM image. (g) Pore size distribution and cumulative pore volume calculated via the QSDFT method based on a slit/cylindrical pore geometry. Inset: Schematic diagram illustrating the NiN_4_‐embedded micropore architecture that facilitates efficient Cl_2_ confinement, transport and conversion.

Aberration‐corrected scanning TEM (AC‐STEM) was employed to resolve the atomic structure, revealing a carbon matrix that exhibited well‐defined locally curved turbostratic lattice fringes [29]. These fringes possessed an interlayer spacing of ∼ 0.34 nm, which approached the characteristic (002) plane of graphitic carbon (Figure 2f). Within this framework, atomic‐scale bright spots were identified anchored across the curved graphitic layers, confirming the presence of isolated Ni single‐atom sites. By adjusting the defocus, Ni atoms residing at various depths within the curved carbon shells were resolved (Figure S1). This depth‐profiling observation confirmed that the Ni SACs were anchored across a multi‐layered, locally curved graphitic framework, spanning from the exterior surfaces to the internal voids. Such a pervasive distribution coupled the intrinsic catalytic potency of Ni SACs with the spatial confinement of the surrounding micropores, thereby ensuring the efficient and localized conversion of Cl_2_/LiCl. The Ni loading was quantified as 1.07 wt% by inductively coupled plasma optical emission spectroscopy (ICP‐OES), with other non‐metal elements quantified via CHNO analysis (Table S1). TEM imaging and XRD analysis (Figure S2) confirmed the formation of curved, bark‐like wrinkled graphitic domains, a morphology likely resulting from the Ni‐catalyzed transformation of asphaltenes during pyrolysis [30, 31]. The resulting curvature induced σ–π rehybridization, which could modulate the electronic structure of the anchored single‐atom catalysts [32]. Notably, the use of abundant asphaltene feedstock together with a facile ball‐milling process enabled a sustainable and scalable synthesis (Figure S3).

To elucidate the origin of this unique morphology, we conducted a control experiment by impregnating NiPc onto the APC host, followed by a secondary annealing at 800°C. Despite achieving a comparable Ni single‐atom mass loading of 1.14 wt%, the resulting NiN_4_‐APC retained the original amorphous morphology of APC with negligible curved graphitic features (Figure S4). The porous structure of NiN_4_‐cPC was further characterized by N_2_ adsorption/desorption (Figures 2g and S5). Among the fitting pore models, the mixed slit/cylindrical QSDFT model yielded the lowest fitting error of 0.103% (Table S2), consistent with the pore features observed. Quantitative analysis further confirmed this micropore‐dominated texture, yielding a micropore volume of 0.42 cm^3^ g^−1^, which accounted for 50.1% of the total pore volume, much higher than 0.29 cm^3^ g^−1^ of APC and 0.15 cm^3^ g^−1^ of NiN_4_‐APC (Table S1). X‐ray Diffraction (XRD) patterns of APC, NiN_4_‐APC, and NiN_4_‐cPC (Figure S6a) revealed no detectable reflections from crystalline Ni species. Notably, compared to the weak signal observed in APC, NiN_4_‐cPC exhibited a significantly more pronounced and sharper peak at 2θ = 26.4° (indexed to the graphitic 002 plane), indicating that Ni catalyzed the formation of locally ordered graphitic domains. This conclusion was further supported by Raman spectroscopy (Figure S6b): the I_D_/I_G_ decreased to 0.72 for NiN_4_‐cPC, which was lower than that of both NiN_4_‐APC (0.81) and APC (0.87), in line with the Ni/Fe/Co‐mediated graphitization effect [33, 34, 35].

To definitively establish the valence state and local coordination environment of the Ni active sites, XANES and extended EXAFS analyses were performed. The Ni K‐edge XANES spectra revealed that the absorption edges of both NiN_4_‐cPC and NiN_4_‐APC were located at lower energies than those of NiO and close to the reference NiPc (Figure 3a), suggesting that the Ni possessed an oxidation state marginally lower than +2. The k^3^‐weighted fourier transform (FT) EXAFS spectra exhibited a single dominant peak arising from the first‐shell Ni–N coordination (Figure 3b), without detectable Ni–Ni scattering signals. The Ni–N peak of NiN_4_‐cPC was shifted towards a slightly lower radial distance of 1.89 Å compared to 1.91 Å of NiN_4_‐APC, which could be attributed to the turbostratic architecture of the carbon host [36, 37]. Quantitative EXAFS fitting yielded a Ni–N coordination number of approximately 4 for both NiN_4_‐cPC (Figure 3c) and NiN_4_‐APC (Figure S7a and Table S3), consistent with a typical NiN_4_ configuration. This proposed structural model was further supported by the good agreement between experimental XANES spectra (Figures 3d,e, and S7b) and simulations by FDMNES.

**FIGURE 3:**
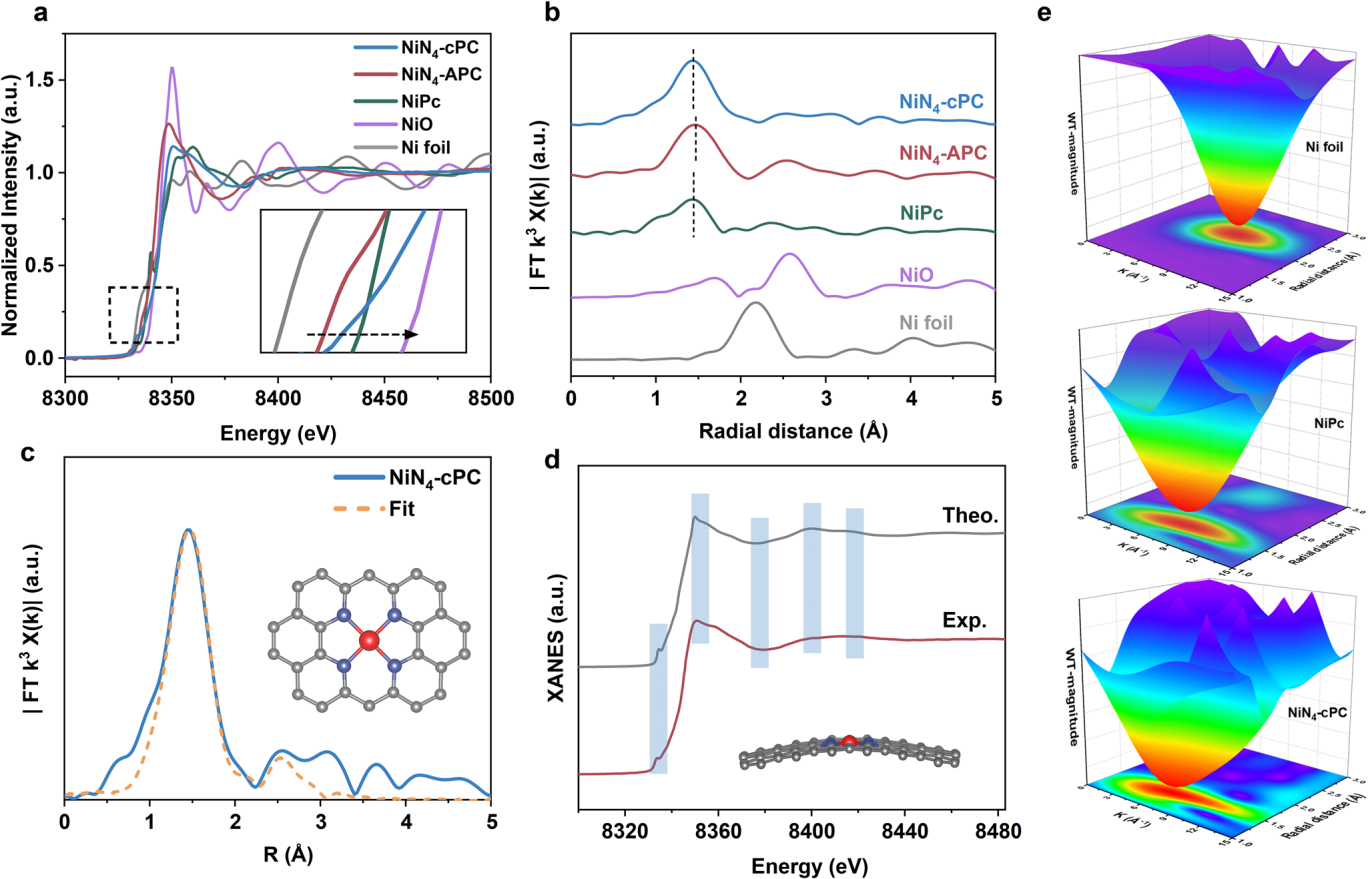
X‐ray absorption spectroscopy characterization of Ni coordination environment. (a) Normalized Ni K‐edge x‐ray absorption near‐edge structure (XANES) spectra, (b) Fourier transform k^3^‐weighted Ni K‐edge extended x‐ray absorption fine structure (FT‐EXAFS) spectra in R space of Ni foil, NiO, NiPc, NiN_4_‐APC, and NiN_4_‐cPC. (c) FT‐EXAFS fitting curves of NiN_4_‐cPC, (d) Comparison between the experimental (Exp.) and theoretical (Theo.) Ni K‐edge XANES spectrum of NiN_4_‐cPC (e) 3D contour wavelet transformed Ni K‐edge EXAFS map of the Ni foil, NiPc, and NiN_4_‐cPC.

Li–Cl_2_ batteries were constructed utilizing the synthesized samples as cathodes and lithium metal as anode. At a current density of 1000 mA g^−1^, the NiN_4_‐cPC and NiN_4_‐APC cathodes delivered remarkable initial deep discharge capacities of 9423 and 7721 mAh g^−1^ (Figure 4a), respectively, with both significantly surpassing the 7163 mAh g^−1^ of the pristine APC. The enhanced initial capacity indicates more extensive LiCl deposition, thereby establishing a substantial initial inventory that served as a vital reservoir for subsequent reversible conversion from LiCl to Cl_2_. The maximum reversible capacity was subsequently quantified employing the same stepwise galvanostatic protocol as shown in Figure 1b. Upon incrementally increasing the charge cutoff capacity from 500 to 2000 mAh g^−1^ at 1000 mA g^−1^, the NiN_4_‐cPC electrode achieved a remarkable reversible capacity of 1822 mAh g^−1^ (Figure 4b). This performance represented an ∼86% enhancement over 980 mAh g^−1^ of pristine APC and substantially outperformed the 1163 mAh g^−1^ of NiN_4_‐APC. Furthermore, while maintaining exceptional cycling stability at a deep discharge capacity of 1500 mAh g^−1^, the Li‐Cl_2_@NiN_4_‐cPC cathode exhibited significantly reduced polarization (Figure S8a). Specifically, it delivered a narrow charge‐discharge voltage gap of 0.53 V, which was markedly lower than that of NiN_4_‐APC (0.64 V) and the pristine APC (0.72 V), accompanied by stable charge‐discharge plateaus. Given the gaseous nature and high diffusivity of the Cl_2_ intermediate, effective confinement of the active species was paramount to mitigating the shuttle effect and ensuring long‐term rechargeability. To rigorously evaluate Cl_2_ retention, the Li–Cl_2_@NiN_4_‐cPC cell was charged to 1500 mAh g^−1^ and subjected to rest periods of varying durations. Remarkably, the subsequent discharge capacities remained ∼1500 mAh g^−1^ after 1, 3, and 12 h of resting, confirming its excellent Cl_2_ retention ability (Figure 4c). Even after a prolonged 42 h rest, the capacity was fully retained and exhibited an apparent increase, despite a slight decrease in the discharge voltage plateau. The voltage evolution was associated with the formation of SO_2_Cl_2_ during retention [5, 6], whereas the increased capacity may be attributed to partial LiCl dissolution, which released occupied pore space and regenerated SOCl_2_ (Figure S9) [4]. Importantly, these resting protocols exerted minimal impact on the overall cycling stability; the rapid recovery of voltage profiles and high‐capacity retention validated the robust Cl_2_ confinement.

**FIGURE 4:**
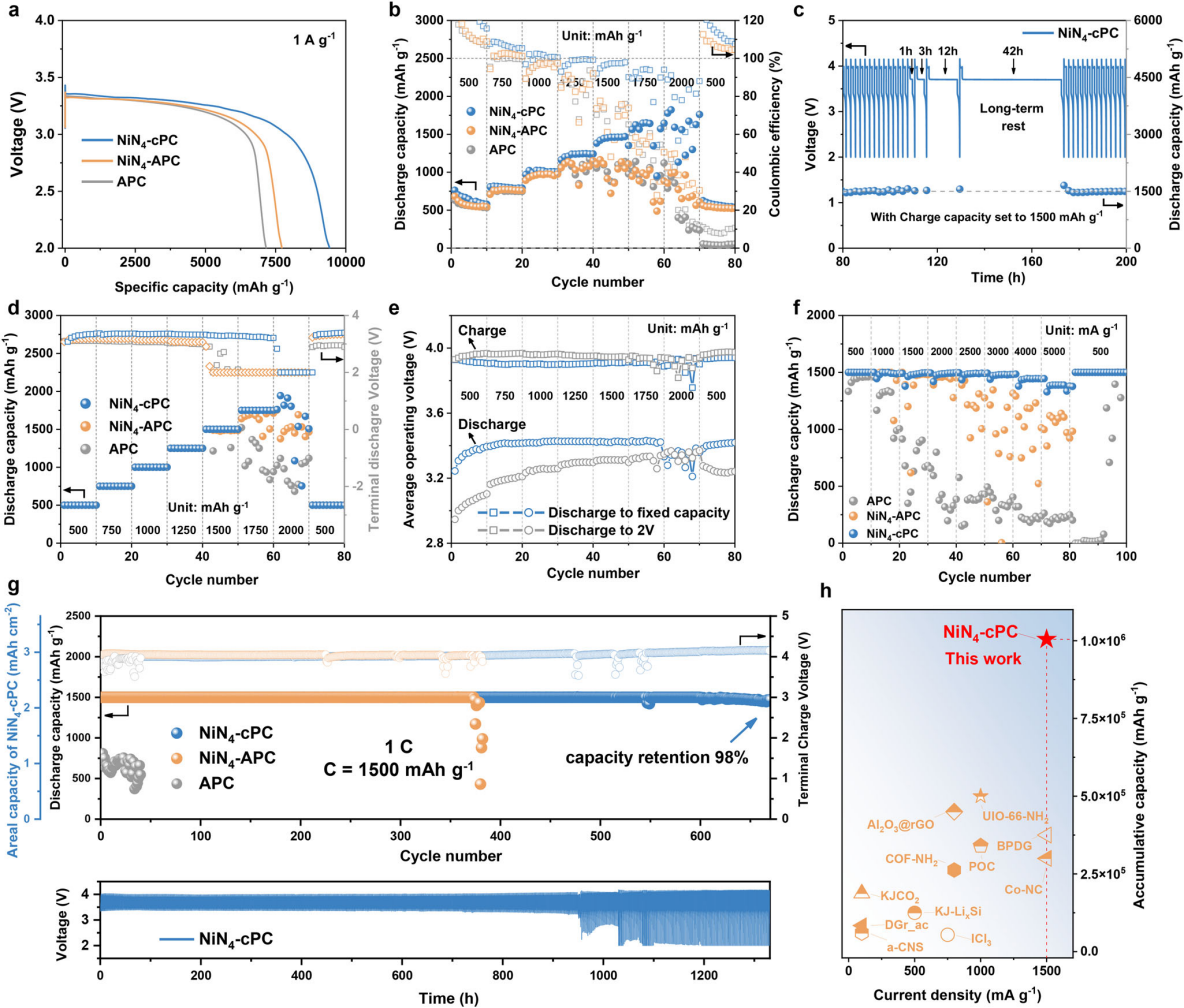
Electrochemical performance of Li‐Cl_2_ Batteries with NiN_4_‐cPC, NiN_4_‐APC, and APC cathodes at room temperature (a) First discharge curves at 1000 mA g^−1^ (b) Evaluation of maximum reversible capacities with different charge cut‐off capacities and 2.0 V cut‐off discharge voltage at 1000 mA g^−1^. (c) Self‐discharge assessment of Li‐Cl_2_@NiN_4_‐cPC battery after resting for varying durations (d) Discharge specific capacity at 1000 mA g^−1^ with equal charge and discharge cutoff capacities. (e) Comparison of average charge/discharge voltage under a curtailed capacity protocol versus the conventional 2.0 V cutoff protocol; (f) Rate capability at a 1500 mAh g^−1^ cutoff capacity for both charge and discharge; (g) Cycling stability at 1500 mA g^−1^ and 1500 mAh g^−1^ with corresponding time‐voltage curve of Li‐Cl_2_@NiN_4_‐cPC (h) Comparison of the cumulative capacity of NiN_4_‐cPC with those of previously reported Li–Cl_2_ batteries.

Notably, all the batteries discussed above were evaluated using the established protocol involving deep discharge to 2.0 V in every cycle. However, such deep discharge often induced severe electrode passivation, as evidenced by the evolution of the charge‐transfer resistance *R*_ct_ extracted from EIS fitting (Figure S10) and prior reports [4, 9]. Analogous to observations in Li–O_2_ and Li–CO_2_ systems [38, 39], this extensive discharge led to the cumulative buildup of insoluble products that clog pores and escalate polarization [9], shortening cell life. To mitigate this irreversibility, we adapted a capacity‐limited cycling strategy from Li‐gas batteries, optimizing it to accommodate the unique features of the Li‐Cl_2_ system. The protocol initiated with a deep first discharge to 2.0 V to establish a sufficient LiCl inventory, followed by cycling with strict capacity limits applied to both charge and discharge. This optimized protocol effectively reduced polarization, decreasing the voltage gap of Li‐Cl_2_@NiN_4_‐cPC from 0.53 to 0.42 V, under the same cut‐off capacity of 1500 mAh g^−1^ and same current density of 1000 mA g^−1^ (Figure S8b). Synergistically, by coupling this protocol with efficient NiN_4_‐cPC catalysis, a remarkable 41.7% polarization reduction from the 0.72 V (catalyst‐free APC) was successfully achieved. During the subsequent long‐term cycling, this approach effectively suppressed polarization growth, enabling the Li‐Cl_2_@NiN_4_‐cPC to maintain significantly lower polarization and superior stability (Figure 4d,e).

The rate capability was further evaluated under a fixed capacity cutoff of 1500 mAh g^−1^ (Figure 4f). Even when the current density was increased to 5000 mA g^−1^, the Li–Cl_2_@NiN_4_‐cPC still exhibited exceptional resilience, retaining a capacity of 1388 mAh g^−1^ (92.5% retention) and high energy efficiency (Figure S11). In stark contrast, the capacities of the control samples, NiN_4_‐APC and APC, declined precipitously to 1140 and 249 mAh g^−1^, respectively. For instance, at 2000 mA g^−1^ and a cutoff capacity of 1500 mAh g^−1^, NiN_4_‐cPC delivered a remarkably low overpotential of 0.62 V, substantially outperforming NiN_4_‐APC and APC, which exhibited overpotentials of 0.71 and 1.83 V, respectively, corresponding to a 1.21 V polarization reduction relative to APC. These results underscored the superior high‐rate tolerance of NiN_4_‐cPC, which was attributed to the sustained acceleration of Cl_2_/LiCl redox kinetics even under rigorous electrochemical loads. The robustness of the cathode was further evaluated via long‐term cycling at 1500 mA g^−1^ with a cut‐off capacity of 1500 mAh g^−1^, translating to an areal capacity of 2.19 mAh cm^−2^. Li–Cl_2_@NiN_4_‐cPC demonstrated extraordinary stability (Figure 4g), sustaining operation for over 670 cycles before the discharge capacity faded to the 98% retention threshold (1470 mAh g^−1^). This translated to a monumental cumulative capacity exceeding 1.0 × 10^6^ mAh g^−1^ (Figure 4h and Table S4), which represented a two‐fold increase over the current state‐of‐the‐art records for Li–Cl_2_ systems [4, 11, 12, 13, 14, 15, 16, 19, 23, 40, 41]. Throughout this extended operational lifespan, the terminal charge potential remained highly stable (e.g., ∼4.12 V at the 600th cycle), indicating minimal polarization growth. The origin of the long‐term stability of Li‐Cl_2_@NiN_4_‐cPC was first investigated by post‐cycling EXAFS and ICP‐OES. NiN_4_‐cPC retained its characteristic Ni–N coordination feature after cycling (Figure S13), while its Ni content remained comparable before and after cycling (1.07 wt% and 1.21 wt%, respectively). Moreover, the fresh and cycled electrolytes exhibited similarly low Ni concentrations of 0.012 and 0.010 ppm, respectively, indicating the stability of the anchored NiN_4_ sites as well. APC, NiN_4_‐APC, and NiN_4_‐cPC were evaluated under the same capacity‐limited protocol, with both charge and discharge capacities fixed at 1500 mAh g^−1^ at 1 C (Figure S12). Although the capacity limitation helped delay excessive LiCl accumulation and electrode passivation, APC and NiN_4_‐APC still exhibited progressively increasing polarization and gradual deterioration of their voltage profiles. In contrast, NiN_4_‐cPC maintained lower polarization and more stable charge and discharge plateaus, indicating that its superior long‐term cycling stability arises from the combined contributions of the spatially coupled cathode architecture and the capacity‐limited cycling protocol.

To evaluate how cathode‐mediated Cl_2_ retention mitigated Li‐anode degradation, the cycled Li anodes paired with NiN_4_‐cPC and APC cathodes were examined. After cycling, the Li anode paired with NiN_4_‐cPC retained a thicker metallic Li layer of approximately 359 µm from the initial 400 µm Li foil, whereas only approximately 310 µm of metallic Li remained in the APC‐paired anode (Figure S14a,b). XPS analysis revealed LiCl‐ and Al–Cl‐related species on both cycled Li surfaces, demonstrating the formation of LiCl‐based interphases (Figure S14c). Consistently, XRD analysis showed weaker LiCl diffraction signals for the NiN_4_‐cPC‐paired anode, indicating reduced LiCl accumulation compared with the APC‐paired anode (Figure S14d). Collectively, this unprecedented combination of ultralong cycle life, deep capacity utilization, and voltage stability attested to the triple synergy of efficient catalysis enabled by active NiN_4_ sites, effective Cl_2_ confinement via the micropore‐dominated architecture, and the structural robustness of the cathode.

Ni K‐edge XANES spectra were collected at representative electrochemical states, including the first discharge to 2 V, charge to 1500 mAh g^−1^, re‐discharge to 1500 mAh g^−1^, and subsequent cycling steps, to investigate the electron transfer process of Ni sites in Li‐Cl_2_@NiN_4_‐cPC. As shown in Figure 5a, the Ni K‐edge absorption edge shifted to markedly lower energy relative to the OCV state during the first discharge process and then shifted back to higher energy upon charging to 1500 mAh g^−1^, although it remained lower than that at OCV, consistent with residual LiCl coverage on the cathode. A similar energy evolution was observed in the second cycle, suggesting an obvious reversible variation in the electronic state of Ni during the Li‐Cl_2_ electrochemical process, which reflected interactions between chlorine‐related species and the Ni sites. In addition, in situ Raman spectroscopy was applied to track the real‐time evolution of reaction intermediates. During the initial discharge (Figure 5b), the characteristic bands of SOCl_2_ at 193, 285, 344, and 490 cm^−1^ diminished or vanished entirely, which was accompanied by the emergence of new signals at 151, 218, and 461 cm^−1^ corresponding to S. This transition was consistent with the cathodic reduction of SOCl_2_ (4Li + 2SOCl_2_ → S + SO_2_ + 4LiCl) [42]. During the subsequent charging step to the cut‐off capacity of 1500 mAh g^−1^, S–Cl intermediates became distinct before being consumed during the subsequent discharge; specifically, the features at 205 cm^−1^ (SCl_2_/S_2_Cl_2_) and 444 cm^−1^ (S_2_Cl_2_) also appeared during charging and subsequently faded during discharging. This behavior pointed to the reaction sequence of S + Cl_2_ → SCl_2_, 2SCl_2_ ↔ S_2_Cl_2_ + Cl_2_ [5], indicating the involvement of electrochemically generated Cl_2_ from LiCl oxidation in the conversion between S and S–Cl species.

**FIGURE 5:**
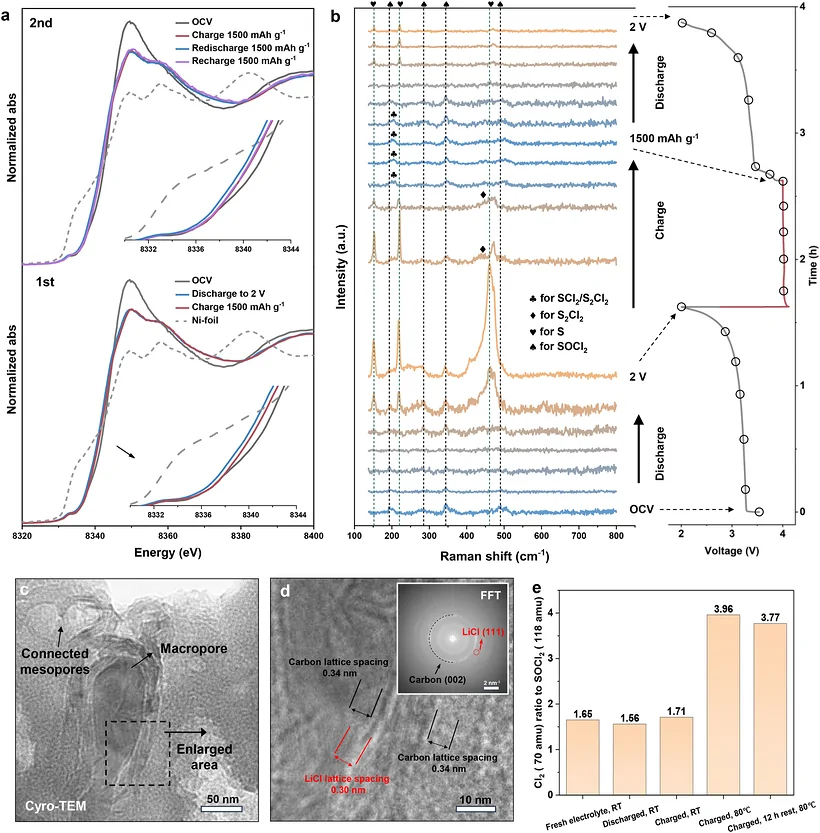
Mechanistic insights into LiCl/Cl_2_ conversion and Cl_2_ retention in NiN_4_‐cPC. (a) In situ Ni K‐edge X‐ray absorption near‐edge structure spectra and (b) in situ Raman spectra with corresponding galvanostatic discharge‐charge curves of Li‐Cl_2_@NiN_4_‐cPC. (c) Ex situ cryo‐HRTEM images of the NiN_4_‐cPC cathode collected in the discharged state, with (d) an enlarged area of the selected region and the corresponding FFT pattern. (e) m/z 70‐to‐m/z 118 signal‐intensity ratios of fresh electrolyte and cycled NiN_4_‐cPC cathodes at different electrochemical states from mass spectrometric.

Cryo‐HRTEM was performed to examine the NiN_4_‐cPC cathode collected in the discharged state after cycling. As shown in Figure 5c, mesopores and macropores within the carbon framework were clearly observed. To distinguish the LiCl lattice from the carbon framework, fast Fourier transform (FFT) analysis was conducted on the enlarged region. The resulting FFT pattern exhibited distinct diffraction spots attributable to crystalline LiCl, together with diffraction rings associated with the carbon framework. Notably, a localized LiCl nanocrystallite with a characteristic size below 10 nm was observed near the pore wall, providing spatial evidence for nanoscale LiCl deposition within the porous cathode structure (Figure 5d). While cryo‐HRTEM provides direct local evidence of LiCl deposition, it probed only a limited region of the cathode. In future studies, small‐angle x‐ray scattering (SAXS) may serve as a complementary technique to examine pore‐structure evolution statistically across broader length scales [43]. Mass spectrometry was employed to probe the release and retention of Cl_2_ from NiN_4_‐cPC cathodes at different electrochemical states (Figures 5e and S15). The fresh electrolyte exhibited an (m/z) 70‐to‐(m/z) 118 signal‐intensity ratio of 1.65, which was taken as the SOCl_2_‐derived background [16]. At room temperature, both the charged and discharged cathodes exhibited ratios close to this baseline value, indicating that the detected signals were dominated by residual SOCl_2_ electrolyte within the cathodes. In contrast, after the charged cathode was heated at 80°C for 30 min, the (m/z) 70‐to‐(m/z) 118 signal‐intensity ratio increased markedly to 3.96, indicating the release of Cl_2_ retained in the charged cathode. After the charged cell was rested at open‐circuit conditions for 12 h, the corresponding ratio decreased only slightly to 3.77 under identical heating conditions. The persistence of this elevated Cl_2_‐related signal supported the effective retention of electrochemically generated Cl_2_ within NiN_4_‐cPC.

Leveraging the recognized durability of Li–SOCl_2_ chemistry across broad temperatures and the strong ambient performance of Li–Cl_2_@NiN_4_‐cPC, we evaluated its behavior over a wide temperature window. The Li–Cl_2_@NiN_4_‐cPC cell was subjected to a stepwise thermal modulation protocol, cycling from 20°C (room temperature) to –40°C and back to +80°C in 10°C increments (Figure 6a). As the temperature decreased to –40°C, we observed a progressive increase in polarization, evidenced by the divergence of terminal charge and discharge voltages (Figure 6b). This phenomenon was attributed to typical kinetic sluggishness, including increased electrolyte viscosity and retarded ion diffusion [18, 23]. Despite the stringent requirements for regulating LiCl deposition and retaining Cl_2_ under such kinetically inhibited conditions, the Li–Cl_2_@NiN_4_‐cPC demonstrated remarkable resilience, sustaining stable cycling for 200 cycles at 1500 mA g^−1^ with a high cutoff capacity of 1500 mAh g^−1^ (Figure 6c). Conversely, elevated temperatures (up to 80°C) surmounted kinetic barriers, resulting in reduced polarization and well‐defined voltage plateaus due to enhanced Li^+^ transport (Figure 6d). However, operating at 80°C triggered a sharp performance decline, which was likely due to the thermal instability of the plastic O‐ring used to seal the coin cell (Figure S16). Demonstrating practical viability, the fabricated pouch cell with a high mass loading of ∼5.1 mg cm^−2^ successfully exhibited a stable open‐circuit voltage of 3.598 V while maintaining stable cycling at a high areal capacity ∼ 7.65 mAh cm^−2^. (Figure 6e,f)

**FIGURE 6:**
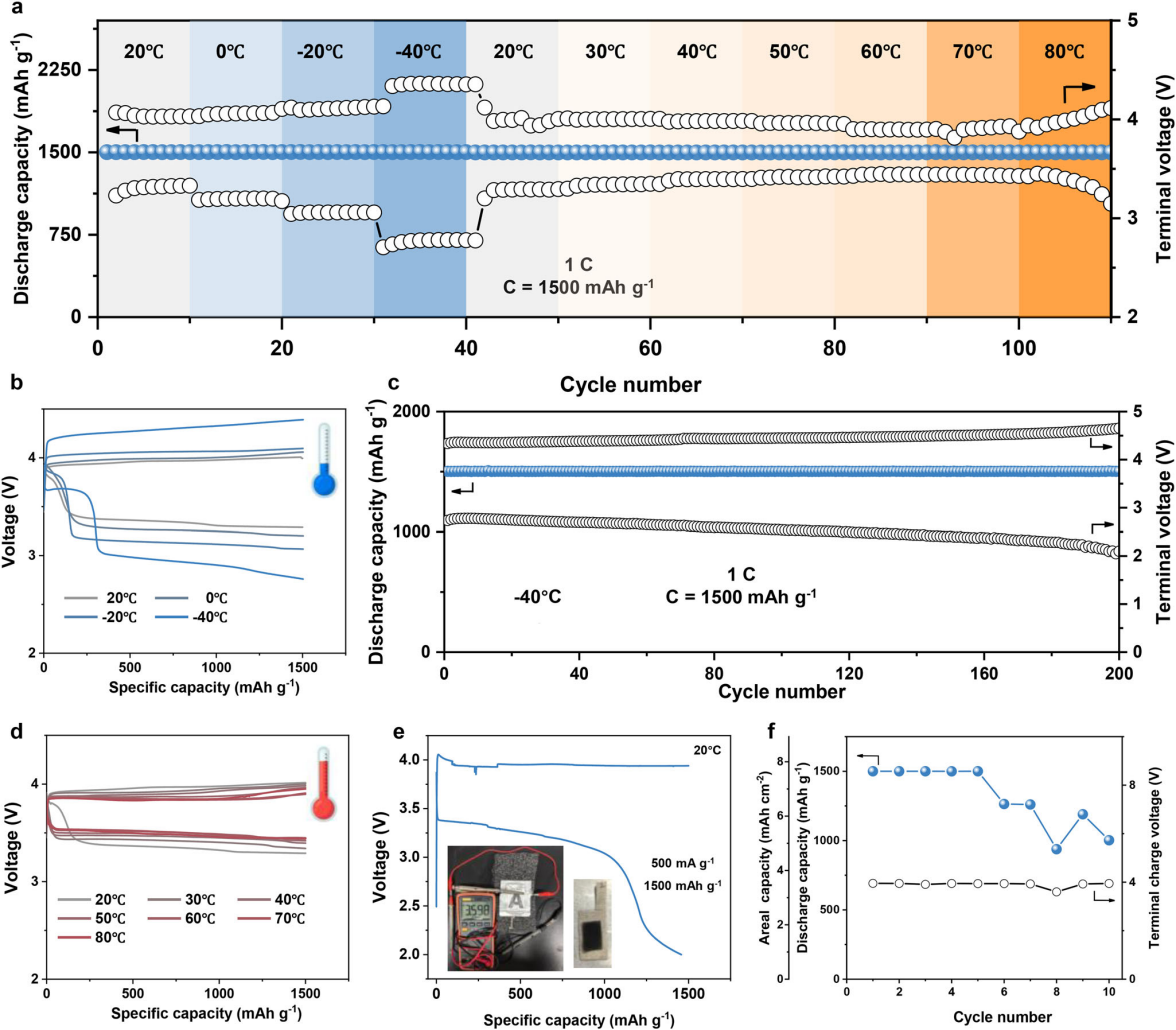
Electrochemical performance of Li‐Cl_2_@NiN_4_‐cPC batteries over a wide temperature range and in a pouch cell configuration (a) Terminal Charge‐discharge voltage and discharge capacities at different temperatures at 1500 mA g^−1^ and 1500 mAh g^−1^ (b) Charge‐discharge curves at temperatures ranging from 20°C to –40°C. (c) Cycling performance at –40°C at 1500 mA g^−1^ and 1500 mAh g^−1^ (d) Charge‐discharge curves at temperatures ranging from 20°C to +80°C. (e) Charge‐discharge curves of a Li–Cl_2_@NiN_4_‐cPC pouch cell at 500 mA g^−1^ and 1500 mAh g^−1^, together with a photograph of the pouch cell containing a high‐loading cathode and (f) cycling performance.

Optimizing Cl_2_ binding strength is essential, as insufficient adsorption not only impedes redox conversion but also permits the escape of weakly bound Cl_2_, causing active‐material loss and aggravating the shuttle effect. The efficacy of Cl_2_ confinement was further corroborated by the reported KBr/methyl orange (MO) oxidation assay [19]. To isolate the contribution of entrapped Cl_2_, charged electrodes underwent a washing step to eliminate loosely adsorbed surface residues. Notably, the charged NiN_4_‐cPC electrode induced a more pronounced bleaching effect than the APC and NiN_4_‐APC electrodes (Figure 7a). These results indicated that the dense microporosity of NiN_4_‐cPC effectively suppressed Cl_2_ effusion. A further control sample, NiN_4_‐APC‐KOH, was prepared by KOH activation and possessed a micropore volume comparable to that of NiN_4_‐cPC, together with atomically dispersed Ni–N sites and a relatively low‐curvature carbon framework (Figure S17 and Table S1).

**FIGURE 7:**
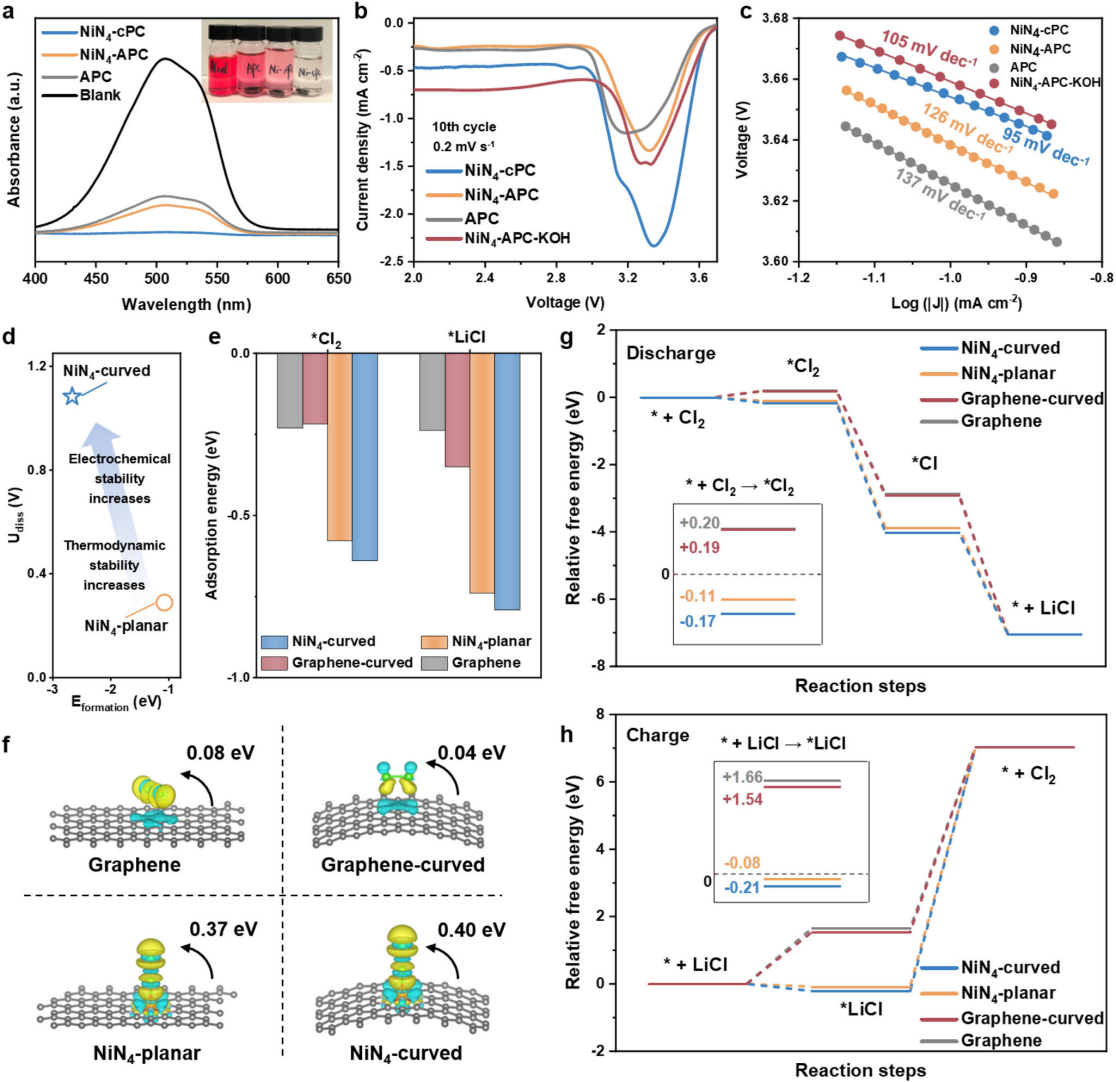
Insights into Cl_2_ retention and Cl_2_/LiCl redox kinetics. (a) UV–vis spectroscopy comparatively assessing the retained Cl_2_ related species in charged APC, NiN_4_‐APC, and NiN_4_‐cPC electrodes, with photographs showing the corresponding color change of the HIn + KBr indicator solution. (b) LSV curves of different Li‐Cl_2_ batteries in the charged state. (c) Tafel plots from LSV curves. (d) Formation energy and Ni dissolution potential (U_diss_) for planar and curved NiN_4_. (e) Adsorption energies of Cl_2_ or LiCl on graphene, curved graphene, planar NiN_4_, and curved NiN_4_ surfaces. (f) Charge density difference of Cl_2_ adsorption (yellow: accumulation; cyan: depletion), with the specific charge transfer calculated by the Bader charge. (g, h) Calculated relative free energy profiles for the discharge and charge pathways on the investigated catalysts, respectively.

Electrochemical evaluation of the charged cells by linear sweep voltammetry (LSV) revealed that Li–Cl_2_@NiN_4_‐cPC exhibited a more positive reduction potential (Figure 7b) and the lowest Tafel slope among the tested samples (Figure 7c), collectively demonstrating the enhanced catalytic activity and favorable reaction kinetics of the curvature‐modulated NiN_4_ sites toward Cl_2_/LiCl‐centered conversion chemistry involving associated redox‐active S–Cl/S–Cl–O species. Li–Cl_2_@NiN_4_‐cPC also delivered the largest cathodic current response, suggesting that the superior Cl^0^‐to‐Cl^−^ conversion kinetics arose from the synergy between the curvature‐modulated reactivity of the NiN_4_ sites and their close spatial proximity to the Cl_2_‐retaining pores. Galvanostatic intermittent titration technique (GITT) measurements were further performed to evaluate the apparent kinetic polarization (Figure S18). Among the four samples, NiN_4_‐cPC exhibited the smallest IR drop of 0.203 V and generally higher apparent Li^+^ diffusion coefficients (D_Li+_), indicating reduced polarization and improved kinetics.

First‐principles DFT calculations were performed to elucidate the combined impact of NiN_4_ catalysis and substrate curvature. A curved NiN_4_ (NiN_4_‐curved) and curved graphene model was constructed alongside a planar counterpart (NiN_4_‐planar and graphene). Thermodynamic evaluations confirmed that both structures satisfied essential stability criteria (*E*_f_ < 0, *U*_diss_ > 0); notably, NiN_4_‐curved exhibited superior robustness (*E*_f_ = −2.66 eV, *U*_diss_ = 1.08 V) compared to the planar model (−1.70 eV, 0.29 V), suggesting enhanced thermodynamic stability under harsh redox conditions (Figure 7d). Regarding the adsorption mechanism, Cl_2_ exhibited low adsorption energies of −0.23 eV on graphene and −0.22 eV on curved graphene (Figures 7e and S19–S22), suggesting negligible binding strength. In stark contrast, the introduction of NiN_4_ single‐atom could provide partially occupied Ni 3d states that effectively hybridized with the σ* antibonding orbital of Cl_2_. This interaction significantly strengthened chemisorption, increasing the adsorption energy to −0.58 eV for NiN_4_‐planar, which was further enhanced by curvature engineering to reach −0.64 eV. Furthermore, differential charge density analysis revealed that curvature amplified electron transfer from Ni to Cl_2_, activating the Cl_2_ molecule to promote rapid downstream redox reactions (Figure 7f).

To further unravel the thermodynamic origins of the enhanced performance, discharge and charge reaction pathways were simulated (Figure 7g,h). The results revealed that the prohibitive Gibbs free energy change (ΔG) on pristine graphene imposed an intrinsic barrier by the adsorption of Cl_2_, inherently limiting conventional carbon cathodes to primary Li–SOCl_2_ systems. In stark contrast, the introduction of Ni sites fundamentally altered this pathway, significantly reducing ΔG to −0.11 eV, enabling a spontaneous adsorption regime. Notably, the curved geometry further optimized this thermodynamic landscape, lowering ΔG to −0.17 eV. Underpinned by significant electron transfer from the Ni center, the NiN_4_‐curved site effectively activated Cl_2_, thereby expediting its conversion and favoring subsequent conversion. Further calculations involving the chlorine‐containing species SCl_2_, S_2_Cl_2_, and SO_2_Cl_2_ consistently demonstrated that the NiN_4_ reduced the thermodynamic difficulty associated with their conversion (Figures S23–27), while curvature modulation further strengthened this effect. During the subsequent charging process, both NiN_4_ motifs exhibited much stronger affinity for LiCl than graphene. Specifically, the NiN_4_‐curved configuration achieved the highest binding energy of −0.79 eV (versus −0.74 eV for NiN_4_‐planar), resulting in the lowest ΔG of −0.21 eV. This characteristic promoted site‐specific LiCl adsorption to ensure intimate cathode contact, which was critical for minimizing oxidation barriers and expediting the decomposition of insulating discharge products [23].

## 3 Conclusion

In summary, we demonstrated that the key to unlocking the potential of high‐energy Li–Cl_2_ chemistry lay in a spatially coupled architecture. By anchoring NiN_4_ single‐atom sites onto the walls of hierarchical, interconnected pores, we successfully bridged Cl_2_ storage, transfer and conversion within the hierarchical matrix. Crucially, this unique curvature within the partially graphitized scaffold intrinsically enhanced the catalytic activity of NiN_4_ towards Cl_2_/LiCl redox kinetics, delivering a remarkable 1.21 V polarization reduction under deep‐discharge conditions of 1500 mAh g^−1^ cut‐off capacity at 2000 mA g^−1^. Concurrently, the robust, partially graphitized support ensured structural integrity, while an optimized capacity‐limited cycling protocol preserved electrode stability. Empowered by these features, the Li–Cl_2_ battery effectively mitigated long‐term cycling degradation, delivering a record‐breaking cumulative capacity exceeding 1.0 million mAh g^−1^ even under stringent high‐rate (1500 mA g^−1^) and deep‐discharge (1500 mAh g^−1^) conditions. Furthermore, its exceptional all‐climate robustness (–40°C to +70°C) and the realization of a 7.65 mAh cm^−2^ areal capacity in pouch cells collectively established a new paradigm for practical, high‐energy storage systems. These results confirmed the exceptional promise of Li–Cl_2_ chemistry and laid the foundation for next‐generation applications.

## Author Contributions

**Minggang Xie**: conceptualization, investigation, methodology, software, data curation, writing – review and editing, writing – original draft, visualization, validation. **Keren Jiang**: methodology. **Xuehai Tan**: data curation. **Xuesong Xie**: conceptualization, methodology. **Zheng Cheng**: conceptualization, software, methodology. **Xianbin Meng**: data curation. **Yuxuan Xue**: data curation. **Yi Guan**: data curation. **Zhixuan Chen**: data curation. **Yifan Li**: data curation. **Renfei Feng**: investigation, methodology, resources. **Zhi Li**: writing – review and editing, funding acquisition, investigation, conceptualization.

## Conflicts of Interest

The authors declare no conflicts of interest.

## Supporting information




**Supporting File**: anie73986‐sup‐0001‐SuppMat.docx.

## Data Availability

The data that support the findings of this study are available from the corresponding author upon reasonable request.
