# Supplementary material for: Spatially Coupled Cl2 Confinement and Activation at Curved Ni Single‐Atom Sites for Highly Reversible Li–Cl2 Batteries
Source: Angew Chem Int Ed Engl. 2026 Jul 31;65(40):e6857946. doi: 10.1002/anie.6857946 (PMC13618270; doi:10.1002/anie.6857946)
Supplement: Supplementary file 1 [file ANIE-65-e6857946-s001.docx]

**Supporting information**

Spatially Coupled Cl_2_ Confinement and Activation at Curved Ni Single-Atom Sites for Highly Reversible Li–Cl_2_ Batteries

Minggang Xie^[a]^, Keren Jiang^[a]^, Xuehai Tan^[a]^, Xuesong Xie^[a]^, Zheng Cheng^[b]^, Xianbin Meng^[a]^, Yuxuan Xue^[a]^, Yi Guan^[a]^, Zhixuan Chen^[a]^, Yifan Li^[a]^, Renfei Feng^[c]^ and Zhi Li*^[a]^

[a]	M. Xie, K. Jiang, X. Tan, X. Xie, X. Meng, Y. Xue, Y. Guan, Z. Chen, Y. Li, Prof. Zhi Li

Department of Chemical and Materials Engineering, University of Alberta, Edmonton, Alberta T6G 1H9, Canada

E-mail: zhi.li@ualberta.ca

[b]	Z. Cheng

State Key Laboratory of Supramolecular Structure and Materials, Jilin University, Changchun 130015, China

[c]	R. Feng

Canadian Light source (CLS), Saskatoon, Saskatchewan S7N 2V3, Canada

Contents:

Experimental and Theoretical calculations

Supplementary Figure 1-27

Supplementary Table 1-4

Reference

## Experimental

**Materials.** C5 asphaltene (n-pentane precipitated asphaltene; Murphy Oil, USA) was used as received. Sodium carbonate (Na_2_CO_3_, Thermo Fisher Scientific, 98%), hydrochloric acid (Thermo Fisher Scientific), nickel phthalocyanine (NiPc, ACROS Organics, 95%), toluene (Thermo Fisher Scientific, ≥98%), thionyl chloride (SOCl_2_, Sigma-Aldrich, 99%), aluminum chloride (AlCl_3_, Sigma-Aldrich, 98%), lithium bis(trifluoromethanesulfonyl)imide (LiTFSI, Sigma-Aldrich, 99%), lithium bis(fluorosulfonyl)imide (LiFSI, Sigma-Aldrich, 99%) were purchased and used as received. Li metal disks, Ni foam and polytetrafluoroethylene (PTFE) were purchased from Canrud Industrial and used as received.

**Synthesis of APC, NiN_4_-cPC, NiN_4_-APC and NiN_4_-APC-KOH.** APC was prepared by ball-milling 2 g asphaltene, 10 g Na_2_CO_3_, and 2 mL toluene at 200 rpm for 8 h, followed by annealing at 800 °C for 2 h under Ar, acid leaching at 80 °C overnight and thorough water washing. NiN_4_-cPC was synthesized using the identical protocol, except that 0.2 g NiPc was added to the initial mixture. The NiN_4_-APC was prepared by impregnating NiPc on APC via rotary evaporation, followed by pyrolysis at 800 °C under Ar for 2 h and subsequent acid washing. To prepare NiN_4_-APC-KOH, APC was first activated with KOH at 700 °C for 0.5 h under an Ar atmosphere. The resulting activated carbon then underwent the identical NiPc impregnation, pyrolysis, and acid-washing procedures as NiN_4_-APC.

**Li-Cl_2_ cell assembly.** The active material powder and polytetrafluoroethylene (PTFE) were mixed at a mass ratio of 9:1 and dispersed in ethanol and sonicated for 1 h. The mixture was subsequently drop-cast onto a circular nickel foam substrate (diameter: 1.2 cm) under 80 ℃ hot plate as cathode. The resulting mass loading of the active material was ~1.5 mg cm^-2^. 2.128 g of aluminum chloride (AlCl_3_), 0.536 g of lithium chloride (LiCl), 0.352 g LiTFSI, and 0.352 g LiFSI were mixed with 8 mL of thionyl chloride (SOCl_2_) and vigorously stirred overnight. The Li-Cl_2_ coin-type battery was assembled in an Ar-filled glovebox with O_2_ and H_2_O concentrations under 2 ppm. Lithium foil was used as anode, and a volume of 120 μL electrolyte was added with a piece of Whatman GF/D glass fiber as separator. High-loading electrodes on Ni foam substrate were fabricated by air-brushing the aforementioned catalyst ink onto Ni foam. NiN_4_-cPC was loaded onto carbon paper for subsequent post-mortem EXAFS and ICP analyses. The pouch-type cell was assembled with a Ni foam/carbon paper supported NiN_4_-cPC cathode (3 cm × 4 cm), a Ni foam supported lithium strip anode (3.5 cm × 4.5 cm), a GF/D separator (4 cm × 5 cm), and same electrolyte as the coin cells. Galvanostatic discharge/charge measurements were conducted using a CT-4008Q-5V50mA-HWX battery testing system (NEWARE). LSV and EIS measurements were performed using a Bio-Logic VSP-3e electrochemical workstation, with the EIS spectra recorded over a frequency range of 100 kHz to 0.01 Hz using an AC amplitude of 10 mV. Galvanostatic intermittent titration technique (GITT) measurements were conducted by executing multiple 20-minute (pulse time, τ) pulsed currents (0.05 A g^−1^) followed by a 120 minutes relaxation period and apparent Li^+^ diffusion coefficients further calculations are performed based on Equation:

$$D^{GITT} = \frac{4}{\pi\tau}({\frac{m_{B}V_{M}}{M_{B}S})}^{2}({\frac{\Delta E_{S}}{\Delta E_{\tau}})}^{2}$$

m_B_ represents the loaded mass of the carbon cathode, while S is the projected geometric area of the cathode (diameter is 12 mm). As a carbon-based approximation, the molar mass (M_B_) is always taken as 12.01 g mol^-1^ for carbon, and the molar volume (V_M_) is estimated to be 5.31 cm^3^ mol^-1^ using a typical density of 2.26 g cm^-3^ for consistency. where τ is the current-pulse duration; ΔE_s_ is the steady-state voltage change between the relaxed potentials before and after the current pulse; and ΔE_τ_ is the voltage change during the current pulse after excluding the instantaneous IR drop.

**Characterizations.** Powder X-ray diffraction (XRD) patterns were recorded using the Bruker D8 diffractometer using Cu Kα-radiation. Raman spectra were recorded on a Renishaw InVIa 365 Raman Instrument with a laser excitation wavelength of 532 nm. Inductively coupled plasma-optical emission spectrometer (ICP-OES) was performed using Thermo Fisher Corp. Registration (N. America). Scanning electron microscopy (SEM) images were obtained on Hitachi S-4800 FESEM. TEM, STEM and the corresponding EDS element mapping images were measured on a JEOL JEM-ARM200CF. X-ray absorption spectroscopy (XAS) spectra were performed at the Shanghai Synchrotron Radiation Facility of BL11b and Very Sensitive Elemental and Structural Probe Employing Radiation from a Synchrotron (VESPERS) at Canadian Light Source. N_2_ adsorption–desorption isotherms (77 K) were measured on Autosorb-iQ-XR and specific BET surface area were determined using the Brunauer–Emmett–Teller (BET) method and pore size distribution analyzed by QSDFT and NLDFT models. UV–vis spectra were recorded using Hitachi U 3900H. The elemental content of the materials was quantified by a Flash 2000 CHNS Analyzer (Thermo Fisher Scientific).

**Cl_2_ retention test.** The methyl orange stock solution was prepared by dissolving 0.1 g of methyl orange in 100 mL of hot water.^[1]^ After cooling, 20 mL of ethanol (95%) was added, and the solution was diluted to 1 L with deionized water. The absorbent working solution was then prepared by diluting 50 mL of the stock solution and 1.0 g of potassium bromide to 500 mL with water. Finally, 400 mL of this mixture was acidified with 100 mL of sulfuric acid (2.57 mol L^-1^) to obtain the final test solution. For the comparative Cl_2_-retention analysis, the fully charged cathode was first rinsed with 1,2-dichloroethane to remove residual electrolyte and dried overnight at room temperature. The dried cathode was then immersed in the prepared absorbent solution and gently agitated. The supernatant was analyzed using a UV-vis spectrophotometer in the wavelength range of 350–650 nm.

**Mass spectrometry measurements.** Mass spectrometry measurements were conducted using a Benchtop Quadrupole Mass Spectrometer for Gas Analysis (HPR-20, Hiden Analytical). Specifically, the charged or discharged electrodes were disassembled in an Ar-filled glovebox and immediately transferred into a sealed glass sample tube connected to the mass spectrometry (HPR-20 from Hiden analysis). The samples were then analyzed under Ar carrier gas or after heating at 80 °C for 30 min.

**Cryo-HRTEM.** For cryo-EM specimen preparation, the cathode powder was uniformly distributed onto a Ni TEM grid as current collector, and the cell was assembled following the standard coin-cell procedure. After 10 cycles, the electrochemical cycling was terminated at the end of discharge. The discharged cathode was gently rinsed with SOCl_2_ and saved in an Ar-filled glovebox for further use. The cryo-EM imaging of the specimens was carried out at the University of Alberta cryo-EM facility, using a Krios G4 300kV cryo-electron microscope equipped with a Selectris-X energy filter and a Falcon 4i direct electron detector. The specimen was prepared at room temperature, and was flash cooled to liquid nitrogen temperature before loading to the microscope. The imaging was carried out at 93 K. The microscope was set at 165 kX magnification, corresponding to a calibrated pixel size of 0.73 Å/pixel. The energy filter slit width was set to 10 eV. The cryo-EM images were recorded in electron counting mode, at a dose rate of 8.1 e/pixel/s, and a total dose of 80 e/Å^2^ were recorded in each movie with a 5.27 s exposure. Motion corrected images were generated by the Thermofisher EPU program.

**In situ Raman.** Experiment was performed on a Raman spectrometer (RENISHAW) with an excitation laser wavelength of 532 nm. The top of the in-situ Raman coin cell was constructed using a quartz window and a PTFE layer with well-sealed.

## Theoretical calculations

First-principles calculations based on density functional theory (DFT) were performed via Vienna Ab-initio Simulation Package (VASP) using projector-augmented wave (PAW) pseudopotentials and Perdew-Burke-Ernzerhof (PBE) functional on Alliance Canada. A cutoff energy of 550 eV was implemented, and the convergence criteria for a 4 × 4 × 1 k-point. The convergence for electronic energy and structural optimization was set to be 1×10^-5^ eV /atom and 0.01 eV/Å, respectively. The long-range van der Waals interactions were treated using the DFT-D3 method. A 20 Å vacuum layer established to mitigate the impact of interlayer interactions.

The formation energy (E_f_) is defined as:

$$E_{f}=E_{catalyst}-E_{substrate}-E_{Ni}$$

And the dissolution potential (U_diss_) is defined as:

$$U_{diss}=U_{diss}\left( bulk_{Ni} \right)-\frac{E_{f}}{2e}$$

The adsorption energy for a Cl_2_ or LiCl molecule on the substrate is calculated using the following formula:

𝐸_𝑎𝑑𝑠_ = 𝐸_𝑡𝑜𝑡𝑎𝑙_ − 𝐸_𝑏𝑎𝑠𝑒_ − 𝐸_adsorbate_

where 𝐸_𝑡𝑜𝑡𝑎𝑙_ is the total energy of the substrate adsorbed with Cl_2_, LiCl or species, while 𝐸_𝑏𝑎𝑠𝑒_ and 𝐸_adsorbate_ are the energies of the clean substrate and the isolated adsorbate, respectively.

The change of Gibbs free energy is calculated based on the discharging-charging process from the reaction sequence:^[2]^

* + Cl_2_ +2Li → *Cl_2_ + 2Li → *Cl + LiCl + Li → LiCl + *LiCl → *+2LiCl

* + S + Cl_2_ +2Li → *S+ Cl_2_ + 2Li → *SCl_2_+ 2Li →*SCl+ LiCl + Li → *S + 2LiCl

* + 2SCl_2_ +2Li → *SCl_2_ + SCl_2_ + 2Li → *S_2_Cl_2_+ Cl_2_+ 2Li →*S_2_Cl+ LiCl + Li + Cl_2_→ *S_2_ + 2LiCl+ Cl_2_

* + SO_2_Cl_2_ +2Li → *SO_2_Cl_2_ + 2Li → *SO_2_Cl+ LiCl+ Li →*SO_2_+ 2LiCl→ *+ SO_2_+ 2LiCl

Figure S1. (a-e) Through-focal AC-STEM image series of NiN_4_-cPC. The appearance of atomic bright spots at different defocus values indicates the distribution of isolated Ni atoms at different depths within the curved carbon matrix.

Figure S2. (a-b) TEM images and (c) XRD pattern of the initial NiN_4_-cPC precursor before thorough acid washing, showing residual Ni clusters that may contribute to catalytic graphitization during pyrolysis.

The enhanced microporosity of NiN_4_-cPC relative to its Ni-free counterpart, APC, likely arises from the combined effects of Ni-assisted carbon etching and structural reorganization during thermal activation. First, dispersed Ni-containing nanoparticles may promote localized carbon gasification through CO/CO_2_-mediated reactions, including Boudouard-type processes, thereby facilitating carbon etching around the Ni species and the formation of additional micropores.^[3,4]^ Second, the Ni-catalyzed development of curved graphitic domains introduces pronounced structural heterogeneity and interfacial mismatch with the surrounding disordered carbon matrix.^[5,6]^ This mismatch may generate cylindrical-like interdomain voids and help preserve the porous framework against collapse during high-temperature annealing. Together, these effects provide a plausible explanation for the higher micropore volume of NiN_4_-cPC and may contribute to the enhanced local confinement of Cl_2_.

Figure S3. Photograph showing the asphaltene precursor, the NiN_4_-cPC product and the 500 mL ball-milling jar.

Figure S4. (a, b) SEM and TEM images of APC, respectively. (c-e) SEM, TEM and AC-STEM images of NiN_4_-APC, respectively.

Figure S5. N_2_ adsorption-desorption plot and pore size distribution of APC, NiN_4_-cPC and NiN_4_-APC. (a) N_2_ adsorption-desorption isotherms. (b,c) QSDFT-fitted pore-size distribution and cumulative pore volume of APC and NiN_4_-APC, respectively, calculated using a slit/cylindrical pore model.

Figure S6. (a) XRD patterns and (b) Raman spectra of APC, NiN_4_-cPC and NiN_4_-APC.

Figure S7. (a) Ni K-edge FT-EXAFS fitting curve and (b) 3D contour wavelet-transform EXAFS map of NiN_4_-APC.

Figure S8. Galvanostatic discharge–charge profiles of NiN_4_-cPC, NiN_4_-APC, and APC under (a) a voltage-limited protocol with discharge to 2 V at 1000 mA g^-1^ and (b) a capacity-limited protocol with both charge and discharge capacities fixed at 1500 mAh g^-1^.

Under otherwise identical electrochemical testing conditions, the electrodes containing NiN_4_ sites exhibited substantially lower and more stable charging plateaus associated with the formation of SCl_2_, S_2_Cl_2_, and SO_2_Cl_2_ (as illustrated in detail in Figure S12b), together with markedly higher corresponding discharge plateaus (as indicated by the red arrows). These changes indicate reduced voltage polarization and improved reversibility of the associated sulfur–chlorine conversion reactions. These trends were further enhanced for the curved configuration.

Figure S9. Galvanostatic discharge–charge profiles of the Li-Cl_2_@NiN_4_-cPC recorded before and after different open-circuit rest periods.

The prolonged retention led to a clear evolution of the discharge profile: the plateau at approximately 3.35 V gradually diminished, whereas a new lower-voltage plateau at approximately 2.9 V became increasingly pronounced. This phenomenon may be associated with the formation of SO_2_Cl_2_ during the prolonged retention,^[7–9]^ which was then reduced according to:

${Cl}_{2}+ {SO}_{2}\to{SO}_{2}{Cl}_{2}$

${SO}_{2}{Cl}_{2}+ 2Li\to{SO}_{2}+2LiCl$ ~ 3.0 V

${Cl}_{2}+ 2Li\to2LiCl$ ~3.5 V

The observed increase in discharge capacity may be attributed to the generation of additional LiCl deposition sites during prolonged retention. Specifically, partial dissolution of LiCl releases previously occupied pore space, enabling further LiCl deposition during the subsequent discharge and corresponding to the regeneration of SOCl_2_^[9]^:

$$LiCl \left( s \right)+ {AlCl}_{4}^{-}\cdot{SOCl}^{+} \left( solv \right)\to{LiAlCl}_{4} \left( \mathrm{solv} \right)+{SOCl}_{2} \left( l \right)$$

$$2SO{Cl}_{2}+ 4Li\to S+{SO}_{2}+4LiCl$$

Figure S10. (a) Nyquist plots and fitted results of the Li–Cl_2_@NiN_4_-cPC recorded at different electrochemical states after a 1 h relaxation period. (b) Equivalent-circuit model and (c) Corresponding fitted charge-transfer resistance (R_ct_).

Figure S11. (a-c) Galvanostatic discharge–charge profiles of NiN_4_-cPC, NiN_4_-APC, and APC at various current densities with a charge and discharge cutoff capacity of 1500 mAh g^-1^, together with their corresponding energy efficiencies.

Figure S12. Selected charge/discharge profiles of (a) NiN_4_-cPC, (c) NiN_4_-APC and (d) APC at 1 C with fixed charge/discharge capacities of 1500 mAh g^-1^. (b) The 100th-cycle NiN_4_-cPC profile selected from (a), with labeled voltage plateaus.

During charging, the major plateau at approximately 3.96 V can be assigned to the oxidation of LiCl to Cl_2_, whereas the subsequent slightly higher-voltage charging region is associated with additional oxidative processes, including the oxidation of SOCl_2_ electrolyte to SO_2_Cl_2_ and the formation of SCl_2_/S_2_Cl_2_, as described by the following reactions^[7,10]^:

$$SO{Cl}_{2}+{AlCl}_{4}^{-}\to{AlCl}_{4}^{-}{SOCl}^{+}+{0.5 Cl}_{2}+e^{-}$$

$$SO{Cl}_{2}+{SOCl}^{+}{AlCl}_{4}^{-} +2{AlCl}_{4}^{-}\to{SCl}^{+}{AlCl}_{4}^{-}+SO_{2}{Cl}_{2}+{Cl}_{2}+2Al{Cl}_{3}+2e^{-}$$

$${Cl}_{2}+{SO}_{2}\to{SO}_{2}{Cl}_{2}$$

$${Cl}_{2}+S\to S{Cl}_{2}$$

$$2S{Cl}_{2}\leftrightarrow S_{2}{Cl}_{2}+{Cl}_{2}$$

During the subsequent discharge process, the initial discharge region with a capacity contribution of less than 55 mAh g^-1^ can be attributed to the consumption/reduction of SCl_2_/S_2_Cl_2_ species. The following discharge plateau at around 3.5 V corresponds primarily to the conversion of Cl_2_ to LiCl, which accounts for up to 86.8% of the total discharge capacity. The final lower-voltage discharge region is assigned to the reduction of SO_2_Cl_2_. As described by the following reactions:

$${2Li}^{+}+\mathrm{SCl}_{2}+{2e}^{-}\to S+2LiCl$$

$${2Li}^{+}+{S_{2}\mathrm{Cl}}_{2}+{2e}^{-}\to2S+2LiCl$$

$${2Li}^{+}+\mathrm{Cl}_{2}+ {2e}^{-}\to2LiCl$$

$${2Li}^{+}+{SO}_{2}{Cl}_{2}+{2e}^{-}\to{SO}_{2}+2LiCl$$

Li–Cl_2_/SOCl_2_ system involves complex multi-species chemistry containing S–Cl/S–Cl–O species. Nevertheless, the quantitative plateau analysis indicates that the Cl_2_/LiCl redox process contributes the dominant fraction of the reversible capacity.

Figure S13. Comparative k^3^-weighted Ni k-edge FT-EXAFS spectra in R space of NiN_4_-cPC in its as-prepared and post-cycling states.

Figure S14. Cross-sectional SEM images and elemental distribution of the cycled Li metal anode after 200 cycles at room temperature for (a) Li-Cl_2_@NiN_4_-cPC and (b) Li-Cl_2_@APC, respectively. (c) XPS and (d) XRD patterns of the corresponding cycled anodes.

Figure S15. Mass-spectrometric analysis of (a) the fresh electrolyte and NiN_4_-cPC cathodes collected in the (b) charged and discharged states at room temperature and (c) after heating at 80 °C for 30 min. (d) Corresponding m/z 70-to-m/z 118 signal-intensity ratios.

Figure S16. Photographs of (a) a freshly assembled coin cell and a coin cell that failed during cycling at 80 °C and (b) an enlarged view of the corroded O-ring of the failed cell.

Figure S17. Characterization of NiN_4_-APC-KOH. (a) XRD pattern, (b) N_2_ adsorption-desorption isotherm, with pore-size distribution inset (c) Ni K-edge FT-EXAFS fitting curve and (d) AC-STEM image.

Figure S18. GITT curves of (a) NiN_4_-cPC, (b) NiN_4_-APC-KOH, (c) NiN_4_-APC and (d) APC, together with (e) the corresponding apparent Li⁺ diffusion coefficients (D_Li+_).

The corresponding apparent Li⁺ diffusion coefficients (D_Li+_) were also estimated, with NiN_4_-cPC generally exhibiting higher values ranging from 2.14×10^−15^ to 1.95×10^−8^ cm^2^ s^−1^. This broad variation in D_Li+_ likely arises from the complex multiphase processes involving pore filling and discharge-product deposition/oxidation within the cathode.

Figure S19. Clean substrate of graphene and optimized adsorption geometries of *Cl, *LiCl and *Cl_2_.

Figure S20. Clean substrate of graphene-curved and optimized adsorption geometries of *Cl, *LiCl and *Cl_2_

Figure S21. Clean substrate of NiN_4_-planar and optimized adsorption geometries of *Cl, *LiCl and *Cl_2_.

Figure S22. Clean substrate of NiN_4_-curved and optimized adsorption geometries of *Cl, *LiCl and *Cl_2_.

Figure S23. Calculated relative free energy profiles of (a) SCl_2_, (b) S_2_Cl_2_ and (c) SO_2_Cl_2_ on the investigated catalysts.

Figure S24. Optimized adsorption geometries of S_x_Cl_y_O_z_ species on graphene.

Figure S25. Optimized adsorption geometries of S_x_Cl_y_O_z_ species on graphene-curved.

Figure S26. Optimized adsorption geometries of S_x_Cl_y_O_z_ species on NiN_4_-planar.

Figure S27. Optimized adsorption geometries of S_x_Cl_y_O_z_ species on NiN_4_-curved.

Table S1. The C, N, and H contents were determined by elemental analysis, whereas the Ni contents were measured by ICP-OES. The BET specific surface areas were calculated using the BET method, and the total and micropore volumes were derived from DFT analysis of the N_2_ adsorption data.

| Samples | C (%) | N (%) | H (%) | Ni (%) | Surface  Area  (m^2^ g^-1^) | Total Pore Volume  (cm^3^ g^-1^) | Micropore Volume (cm^3^ g^-1^) | I_D_/I_G_ |
| --- | --- | --- | --- | --- | --- | --- | --- | --- |
| APC | 82.14 | 0.31 | 0.24 | - | 1078.3 | 0.960 | 0.292 | 0.87 |
| NiN_4_-cPC | 93.07 | 1.11 | 0.49 | 1.07 | 1296.8 | 0.841 | 0.420 | 0.72 |
| NiN_4_-APC | 92.52 | 1.29 | 0.48 | 1.14 | 576.4 | 0.857 | 0.152 | 0.81 |
| NiN_4_-APC-KOH | — | — | — | 1.35 | 1332.7 | 0.801 | 0.44 | — |
| Asphaltene | 80.22 | 1.04 | 8.41 | - | - | - | - | - |

Table S2. Comparison of fitting errors obtained using different QSDFT and NLDFT models for NiN_4_-cPC.

| Models | slit/cylindr. pores, QSDFT | slit pore, QSDFT | cylindr/sphere pores, QSDFT | slit/cylindr/sphere pores, QSDFT | slit pore, NLDFT | cylindr. pores, NLDFT | slit/cylindr. pore, NLDFT |
| --- | --- | --- | --- | --- | --- | --- | --- |
| Fitting error | 0.103 % | 0.426 % | 1.136 % | 1.059 % | 1.052 % | 2.683 % | 2.761 % |

Table S3. Ni K-edge EXAFS fitting parameters.

S_0_^2^ = 0.7676 was obtained by Ni foil (as reference) fitting.

| Samples | Scattering  pair | CN | R (Å) | σ_2_ (10^-3^Å^2^) | △E_0_ (eV) | R-factor |
| --- | --- | --- | --- | --- | --- | --- |
| Ni foil | Ni-Ni | 12 | 2.485 | 5.96 | 6.95 | 0.002 |
| NiN_4_-cPC | Ni-N  Ni-C1  Ni-C2 | 3.87  4.97  0.20 | 1.89  2.63  2.85 | 6.96  6.97  7.71 | 1.12 | 0.019 |
| NiN_4_-APC | Ni-N | 4.04 | 1.91 | 1.93 | 8.62 | 0.017 |
| NiN_4_-APC-KOH | Ni-N | 4.39 | 1.92 | 1.68 | -3.161 | 0.015 |

Table S4. Comparison of the electrochemical performance of the Li-Cl_2_ cell in this work with that of previously reported rechargeable chlorine-based battery systems.

| Cathodes with Li/Na-Cl_2_ battery | Current density  (mA g^-1^) | Reversible capacity  (mAh g^-1^) | Cycle  number | Cumulative capacity  (mAh g^-1^) | Operating temperature range  (℃) | Reference |
| --- | --- | --- | --- | --- | --- | --- |
| Li−Cl_2_@NiN_4_-cPC | **1500** | **1500** | **670** | **1,004,600** | **-40 to 70** | **This work** |
| Li−Cl_2_@KJCO_2_ | 100 | 1200  2500  4000 | 130  75  7 | ~ 156,000  ~ 187,500  ~ 28,000 | -80 to RT | Ref^[11]^ |
| Li−Cl_2_@POC | 200,500charge;  1000discharge | 2000 | 170 | ~ 340,000 | -40 to RT | Ref^[12]^ |
| Li−Cl_2_@UIO-66-NH_2_ | 1000 | 1000 | 500 | ~ 500,000 | N/A | Ref^[13]^ |
| Li−Cl_2_@COF-NH_2_ | 800 | 2500 | 105 | ~ 262,500 | -40 to RT | Ref^[14]^ |
| Li−Cl_2_@Co-NC | 1500 | 500 | 600 | < 300,000 | -40 to RT | Ref^[2]^ |
| Li-Cl_2_@Al_2_O_3_@rGO | 800 | 2500 | 180 | ~ 450,000 | -40 to RT | Ref^[15]^ |
| Li-Cl_2_@BPDG | 1500 | 500 | 750 | ~ 375,000 | N/A | Ref^[1]^ |
| Li-Cl_2_@DGr_ac | 100 | 600 | 140 | 84,000 | N/A | Ref^[16]^ |
| Li-ICl_3_ | 750 | ≤271 | 200 | < 54,200 | N/A | Ref^[17]^ |
| Li-Cl_2_@a-CNS | 100 | 500 | 120 | ~60,000 | N/A | Ref^[9]^ |
| Li-Cl_2_@Li_x_Si | 500 | 500 | 250 | ~ 125,000 | -40 to RT | Ref^[18]^ |
| I_2_-assisted Li-SOCl_2_ | 5 mA cm^-2^ | 6 mAh cm^-2^ | 50 | - | N/A | Ref^[19]^ |

## Reference

[1]	J. Han, X. Wei, G. Ma, H. Wang, Y. Sun, Z. Liu, L. Zhao, C. Ma, Q. Liu, W. Feng, D. Kong, W. Lv, Q.-H. Yang, L. Zhi, “Blocking pore design enables highly reversible lithium–chlorine batteries” *Energy Environ. Sci.* **2025**, *18*, 8052–8065.

[2]	P. Li, C. Ma, Y. Wang, S. Zhai, G. Ma, D. Kong, Z. Li, “Co Single-Atom Catalysis for High-Efficiency LiCl/Cl2 Conversion in Rechargeable Lithium-Chlorine Batteries” *Advanced Materials* **2025**, *37*, 2418990.

[3]	Y. K. Han, C.-I. Ahn, J.-W. Bae, A. R. Kim, G. Y. Han, “Effects of Carbon Formation on Catalytic Performance for CO2 Reforming with Methane on Ni/Al2O3 Catalyst: Comparison of Fixed-Bed with Fluidized-Bed Reactors” *Ind. Eng. Chem. Res.* **2013**, *52*, 13288–13296.

[4]	Z. Bian, S. Das, M. H. Wai, P. Hongmanorom, S. Kawi, “A Review on Bimetallic Nickel-Based Catalysts for CO2 Reforming of Methane” *ChemPhysChem* **2017**, *18*, 3117–3134.

[5]	S. Khoshk Rish, A. Tahmasebi, R. Wang, J. Dou, J. Yu, “Formation mechanism of nano graphitic structures during microwave catalytic graphitization of activated carbon” *Diamond and Related Materials* **2021**, *120*, 108699.

[6]	Y. Liu, Q. Liu, J. Gu, D. Kang, F. Zhou, W. Zhang, Y. Wu, D. Zhang, “Highly porous graphitic materials prepared by catalytic graphitization” *Carbon* **2013**, *64*, 132–140.

[7]	P. Liang, G. Zhu, W. Wang, C.-L. Huang, S.-C. Wu, J. Zhou, X. Zhou, Y. Wu, S. Wang, M. Wang, L. Zhang, C. C. Ming, J. Li, F. Wang, M. Sun, Y.-Y. Li, B.-J. Hwang, H. Dai, “Carbon Nanotubes for Rechargeable Na/Cl2 Batteries” *J. Am. Chem. Soc.* **2025**, *147*, 18541–18549.

[8]	X. Zhao, M. Liao, S. Geng, Y. Wang, S. Wang, Z. Ouyang, Q. Xu, B. Yuan, C. Zhang, S. Tang, L. Ye, L. Wu, H. Peng, H. Sun, “Synergistic sulfur-chlorine battery chemistry towards efficient energy storage” *Nat Commun* **2026**, *17*, 3088.

[9]	G. Zhu, X. Tian, H.-C. Tai, Y.-Y. Li, J. Li, H. Sun, P. Liang, M. Angell, C.-L. Huang, C.-S. Ku, W.-H. Hung, S.-K. Jiang, Y. Meng, H. Chen, M.-C. Lin, B.-J. Hwang, H. Dai, “Rechargeable Na/Cl2 and Li/Cl2 batteries” *Nature* **2021**, *596*, 525–530.

[10]	G. Zhu, H. Xu, S. Wang, Y. Zhang, J.-H. Fang, T. Dai, D. J. Zheng, W. Chen, H. Roh, D. Wang, D. Menga, S. Yu, H. Iriawan, Y. Shao-Horn, J. Li, “Increasingly Reversible Na/Cl2 and Li/Cl2 Batteries” *J. Am. Chem. Soc.* **2026**, *148*, 22673–22686.

[11]	P. Liang, G. Zhu, C.-L. Huang, Y.-Y. Li, H. Sun, B. Yuan, S.-C. Wu, J. Li, F. Wang, B.-J. Hwang, H. Dai, “Rechargeable Li/Cl2 Battery Down to −80 °C” *Advanced Materials* **2024**, *36*, 2307192.

[12]	Y. Xu, S. Zhang, M. Wang, Y. Meng, Z. Xie, L. Sun, C. Huang, W. Chen, “Enrichment of Chlorine in Porous Organic Nanocages for High-Performance Rechargeable Lithium–Chlorine Batteries” *J. Am. Chem. Soc.* **2023**, *145*, 27877–27885.

[13]	Y. Xu, L. Jiao, J. Ma, P. Zhang, Y. Tang, L. Liu, Y. Liu, H. Ding, J. Sun, M. Wang, Z. Li, H.-L. Jiang, W. Chen, “Metal-organic frameworks for nanoconfinement of chlorine in rechargeable lithium-chlorine batteries” *Joule* **2023**, *7*, 515–528.

[14]	Y. Xu, M. Wang, M. Sajid, Y. Meng, Z. Xie, L. Sun, J. Jin, W. Chen, S. Zhang, “Organocatalytic Lithium Chloride Oxidation by Covalent Organic Frameworks for Rechargeable Lithium-Chlorine Batteries” *Angewandte Chemie International Edition* **2024**, *63*, e202315931.

[15]	Z. Yang, Y. Huang, W. Zhou, H. Fan, Z. Ding, X. Yan, Y. Lu, A. S. Sigov, W. Huang, L. Gao, C. Huang, “High-Performance Rechargeable Lithium-Chlorine Batteries with ALD Conformal Starburst Porous Graphene Positive Electrodes” *Advanced Science* **2025**, *12*, e03113.

[16]	G. Zhu, P. Liang, C.-L. Huang, C.-C. Huang, Y.-Y. Li, S.-C. Wu, J. Li, F. Wang, X. Tian, W.-H. Huang, S.-K. Jiang, W.-H. Hung, H. Chen, M.-C. Lin, B.-J. Hwang, H. Dai, “High-Capacity Rechargeable Li/Cl2 Batteries with Graphite Positive Electrodes” *J. Am. Chem. Soc.* **2022**, *144*, 22505–22513.

[17]	P. Li, X. Li, Y. Guo, A. Chen, R. Zhang, Y. Hou, Q. Xiong, Y. Wang, Z. Chen, J. Zhu, M. Zhu, C. Zhi, “Development of an energy-dense and high-power Li-Cl2 battery using reversible interhalogen bonds” *Chem* **2024**, *10*, 352–364.

[18]	B. Yuan, L. Wu, S. Geng, Q. Xu, X. Zhao, Y. Wang, M. Liao, L. Ye, Z. Qu, X. Zhang, S. Wang, Z. Ouyang, S. Tang, H. Peng, H. Sun, “Unlocking Reversible Silicon Redox for High-Performing Chlorine Batteries” *Angewandte Chemie International Edition* **2023**, *62*, e202306789.

[19]	G. Chen, W. Li, X. Du, C. Wang, X. Qu, X. Gao, S. Dong, G. Cui, L. Chen, “Transforming a Primary Li-SOCl2 Battery into a High-Power Rechargeable System via Molecular Catalysis” *J. Am. Chem. Soc.* **2023**, *145*, 22158–22167.
